# Supplementary material for: Clinical Characteristics and Epidemiological Features of Hepatitis E Virus Infection Among People Living with HIV in Shanghai, China
Source: Viruses. 2025 Jul 25;17(8):1038. doi: 10.3390/v17081038 (PMC12390178; doi:10.3390/v17081038)
Supplement: Supplementary file 1 [file viruses-17-01038-s001.zip › viruses-3734059-Supplementary Materials.pdf]

Table S1. List of primers used for HEV RNA quantification [1].

Primer	Sequence (5' to 3')	Application
HEV-F	GGTGGTTTCTGGGGTGAC	RT-qPCR assays
HEV-R	AGGGGTTGGTTGGATGAA	
HEV-Probe	FAM-TGATTCTCAGCCCTTCGC-MGB	

HEV, Hepatitis E Virus; F, Forward primer; R, Reverse primer; FAM, 6-carboxyfluorescein; MGB, Minor groove binder; RT-qPCR, reverse transcription-quantitative polymerase chain reaction.

Table S2. Published studies on HEV and HIV coinfection.

Research Period	Country	EIA assay employed	Anti-HEV IgG positive, n%	Anti-HEV IgM positive, n%	Risk factors with HEV infection	HEV RNA positive, n(%)	Chronic HEV infection	References
1985–2009	USA	Nizhniy Novgorod	6.7% (13/194)		HIV Viral load, CD4 counts	0	0	[2]
1994-1995	Argentin	Abbott laboratories	6.6% (32/484)		NS	NR	0	[3]
2001	Zambia	Fortress Diagnostics Ltd	71% -adults (22/31)	0	NS	NR	0	[4]
2001– 2002	Italy	DSI	19.4% (14/72)	0	NS	NR	0	[5]
2003-2014	USA	Wantai	19% (32/166)	0.6% (1/166)	NS	0	0	[6]
2004-2009	Australia	Wantai	6.3% (12/191)		NS	2(1.0%)	0	[7]
2005-2008	France	Adaltis	3.7% (4/108)	0.9% (1/108)	NS	1(0.9%)	0	[8]
2006-2013	Cambodia	Wantai	30.1% (248/825)	1.1% (9/825)	NS	0	0	[9]
2008	France	Adaltis	1.5% (4/261)	0	NS	0	0	[10]
2008	Switzerland	Adaltis	2.6% (19/735)	Unknown	CD4 account	1(0.1%)	1(0.1%)	[11]
2008-2010	Ghana	Wantai	45.3% (182/402)	0.7% (3/402)	NS	0	0	[12]
2009	Germany	Wantai	26% (63/246)	NS	NS	0	0	[13]
		MP Biomedicals	1.6% (4/246)					
2009	France	Nizhniy	7.3%	1.2%	CD4	2 (0.8%)	0	[14]

		Novgorod	(18/245)	(5/245)				
2009	Spain	Wantai	26% (161/613)	ND	Age, Male sex	1(0.2%)	0	[15]
2009-2010	Cameroon	Wantai	14.2% (41/289)	0	NS	0	0	[12]
2009-2010	England (Southwest)	Wantai	9.4% (13/184)	0.5% (1/184)	consumption of raw pork	0	0	[16]
2009-2010	China	Wantai	39.4% (252/639)	0.3% (2/639)	Age,gender,ethnicity, region	0	0	[17]
2010	Argentina	Diapro	7.35% (15/204)	2.45% (5/204)	CD4 counts	1 (0.5%)	0	[18]
2011	Zambia	Fortress Diagnostics Ltd	16% -children (31/194)	0	NS	NR	0	[4]
2006- 2011	Holland	Wantai	11.7% (30/256)	0	NS	NR	0	[19]
2010-2011	Italy(Foggia)	DSI	2% (2/100)	1% (1/100)	NS	1(1%)	0	[20]
2011	Spain	Bioelisa	9% (22/238)	0	Cirrhosis	3 (1.2%)	0	[21,22]
2011-2013	Croatia	RecomLine Kits	1.1% (1/88)	12.5% (11/88)	NS	0	0	[23]
2007-2013	Brazil	RecomLine Kits	10.7% (38/354)	1.4% (5/354)	NS	0	0	[24]
2012	Iran	Diapro	10% (10/100)	0	NS	0	0	[25]
2012- 2013	Spain	Wantai	9.8% (88/894)		Age	5(0.6%)	0	[26]
2012-2013	Brazil	Mikrogen Diagnostik	6.7% (24/360)	0.8% (3/360)	Age,Marital status,Multiple partners,Sanitation, Alcohol use	8 (2.2%)	0	[27]
2013	Italy	DSI	6.7% (34/509)	0.9% (5/509)	Cirrhosis	5(0.9%)	0	[28]
2014	Scotland	Mikrogen Diagnostik	1.0% (1/99)	1.0% (1/99)	NS	NR	0	[29]
2015-2016	China (Yunnan province)	Wantai	44.42% (342/770)	0.78% (6/770)	Age, gender, CD4 counts,WHO stage, marital status, TC levels	0	0	[30]
2017	Fars Province (southern	Diapro	10.4% (26/251)	NS	NS	NR	0	[31]

	Iran)							
2020-2021	Greece	Mikrogen Diagnostik	16.5% (115/696)	8.6% (60/696)	HAV infections	16 (2.3%)	0	[32]
2023	Southwest Cameroon	Wantai	7.3% (17/233)	2.6% (6/233)	NS	0	0	[33]
NR	Greece	Adaltis	7.3% (18/243)	ND	Age	NR	0	[34]
NR	France	Adaltis	4.4% (8/184)	1.6% (3/184)	NS	1 (0.5%)	1 (0.5%)	[35]
NR	Spain	Diapro	10.4% (45/448)	0.7% (3/448)	NS	2(0.4%)	0	[36]
NR	Nigeria	Diagnosis Automation Inc	30.0% (24/80)	1.3% (1/80)	NS	0	0	[37]
NR	Gabón	GeneLabs	7.1% (13/183)	0	HIV Viral load	NR	0	[38]
NR	Malaysia	Abbott laboratories	10.3% (15/145)	4.1% (6/145)	NS	NR	0	[39]

HEV, Hepatitis E Virus; BMI, Body Mass Index; ALT, Alanine Aminotransferase; TC,Total Cholesterol; EAI, Enzime Immunoassay; IgG, Immunoglobulin G; IgM, Immunoglobulin M; U.S.A., United State of America; ART, antiretroviral therapy; NR, Not Reported; NS, Non-significant;

References

1. Wang, B.; Harms, D.; Papp, C.P.; Niendorf, S.; Jacobsen, S.; Lütgehetmann, M.; Pischke, S.; Wedermeyer, H.; Hofmann, J.; Bock, C.T. Comprehensive Molecular Approach for Characterization of Hepatitis E Virus Genotype 3 Variants. *J. Clin. Microbiol.* **2018**, 56, doi:10.1128/jcm.01686-17.
2. Crum-Cianflone, N.F.; Curry, J.; Drobeniuc, J.; Weintrob, A.; Landrum, M.; Ganesan, A.; Bradley, W.; Agan, B.K.; Kamili, S. Hepatitis E virus infection in HIV-infected persons. *Emerg. Infect. Dis.* **2012**, *18*, 502-506, doi:10.3201/eid1803.111278.
3. Fainboim, H.; González, J.; Fassio, E.; Martínez, A.; Otegui, L.; Eposto, M.; Cahn, P.; Marino, R.; Landeira, G.; Suaya, G.; et al. Prevalence of hepatitis viruses in an anti-human immunodeficiency virus-positive population from Argentina. A multicentre study. *J. Viral. Hepat.* **1999**, *6*, 53-57, doi:10.1046/j.1365-2893.1999.t01-1-6120135.x.
4. Jacobs, C.; Chiluba, C.; Phiri, C.; Lisulo, M.M.; Chomba, M.; Hill, P.C.; Ijaz, S.; Kelly, P. Seroepidemiology of hepatitis E virus infection in an urban population in Zambia: strong association with HIV and environmental enteropathy. *J. Infect. Dis.* **2014**, *209*, 652-657, doi:10.1093/infdis/jit409.
5. Rapicetta, M.; Monarca, R.; Kondili, L.A.; Chionne, P.; Madonna, E.; Madeddu, G.; Soddu, A.; Candido, A.; Carbonara, S.; Mura, M.S.; et al. Hepatitis E virus and hepatitis A virus

exposures in an apparently healthy high-risk population in Italy. *Infection.* **2013**, *41*, 69-76, doi:10.1007/s15010-012-0385-8.
6. Sherman, K.E.; Terrault, N.; Barin, B.; Rouster, S.D.; Shata, M.T. Hepatitis E infection in HIV-infected liver and kidney transplant candidates. *J. Viral. Hepat.* **2014**, *21*, e74-77, doi:10.1111/jvh.12233.
7. Yong, M.K.; Paige, E.K.; Anderson, D.; Hoy, J.F. Hepatitis E in Australian HIV-infected patients: an under-recognised pathogen? *Sex. Health.* **2014**, *11*, 375-378, doi:10.1071/sh13198.
8. Sellier, P.; Mazeron, M.C.; Tesse, S.; Badsi, E.; Evans, J.; Magnier, J.D.; Sanson-Le-Pors, M.J.; Bergmann, J.F.; Nicand, E. Hepatitis E virus infection in HIV-infected patients with elevated serum transaminases levels. *Virol. J.* **2011**, *8*, 171, doi:10.1186/1743-422x-8-171.
9. Nouhin, J.; Barennes, H.; Madec, Y.; Prak, S.; Hou, S.V.; Kerleguer, A.; Kim, S.; Pean, P.; Rouet, F. Low frequency of acute hepatitis E virus (HEV) infections but high past HEV exposure in subjects from Cambodia with mild liver enzyme elevations, unexplained fever or immunodeficiency due to HIV-1 infection. *J. Clin. Virol.* **2015**, *71*, 22-27, doi:10.1016/j.jcv.2015.07.304.
10. Maylin, S.; Stephan, R.; Molina, J.M.; Peraldi, M.N.; Scieux, C.; Nicand, E.; Simon, F.; Delaugerre, C. Prevalence of antibodies and RNA genome of hepatitis E virus in a cohort of French immunocompromised. *J. Clin. Virol.* **2012**, *53*, 346-349, doi:10.1016/j.jcv.2012.01.001.
11. Kenfak-Foguena, A.; Schöni-Affolter, F.; Bürgisser, P.; Witteck, A.; Darling, K.E.; Kovari, H.; Kaiser, L.; Evison, J.M.; Elzi, L.; Gurter-De La Fuente, V.; et al. Hepatitis E Virus seroprevalence and chronic infections in patients with HIV, Switzerland. *Emerg. Infect. Dis.* **2011**, *17*, 1074-1078, doi:10.3201/eid/1706.101067.
12. Feldt, T.; Sarfo, F.S.; Zoufaly, A.; Phillips, R.O.; Burchard, G.; van Lunzen, J.; Jochum, J.; Chadwick, D.; Awasom, C.; Claussen, L.; et al. Hepatitis E virus infections in HIV-infected patients in Ghana and Cameroon. *J. Clin. Virol.* **2013**, *58*, 18-23, doi:10.1016/j.jcv.2013.05.004.
13. Pischke, S.; Schwarze-Zander, C.; Bremer, B.; Lehmann, P.; Wiegand, S.B.; Gisa, A.; Behrendt, P.; Strassburg, C.P.; Manns, M.P.; Wedemeyer, H.; Rockstroh, J.K. Hepatitis E Virus Seroprevalence Rate in HIV-Infected Patients in Germany: A Comparison of Two Commercial Assays. *Intervirology.* **2015**, *58*, 283-287, doi:10.1159/000441472.
14. Renou, C.; Lafeuillade, A.; Cadranel, J.F.; Pavio, N.; Pariente, A.; Allègre, T.; Poggi, C.; Pénaranda, G.; Cordier, F.; Nicand, E. Hepatitis E virus in HIV-infected patients. *AIDS.* **2010**, *24*, 1493-1499, doi:10.1097/QAD.0b013e32833a29ab.
15. Pineda, J.A.; Cifuentes, C.; Parra, M.; Merchante, N.; Pérez-Navarro, E.; Rivero-Juárez, A.; Monje, P.; Rivero, A.; Macías, J.; Real, L.M. Incidence and natural history of hepatitis E virus coinfection among HIV-infected patients. *AIDS.* **2014**, *28*, 1931-1937, doi:10.1097/qad.0000000000000378.
16. Keane, F.; Gompels, M.; Bendall, R.; Drayton, R.; Jennings, L.; Black, J.; Baragwanath, G.; Lin, N.; Henley, W.; Ngui, S.L.; et al. Hepatitis E virus coinfection in patients with HIV infection. *HIV. Med.* **2012**, *13*, 83-88, doi:10.1111/j.1468-1293.2011.00942.x.

17. Zeng, H.; Wang, L.; Liu, P.; Liao, L.; Wang, L.; Shao, Y. Seroprevalence of hepatitis E virus in HIV-infected patients in China. *AIDS.* **2017**, *31*, 2019-2021, doi:10.1097/qad.0000000000001585.
18. Debes, J.D.; Martínez Wassaf, M.; Pisano, M.B.; Isa, M.B.; Lotto, M.; Marianelli, L.G.; Frassone, N.; Ballari, E.; Bohjanen, P.R.; Hansen, B.E.; Ré, V. Increased Hepatitis E Virus Seroprevalence Correlates with Lower CD4+ Cell Counts in HIV-Infected Persons in Argentina. *PLoS. One.* **2016**, *11*, e0160082, doi:10.1371/journal.pone.0160082.
19. Hassing, R.J.; van der Eijk, A.A.; Lopes, V.B.; Snijdewind, I.J.; de Man, R.A.; Pas, S.D.; van der Ende, M.E. Hepatitis E prevalence among HIV infected patients with elevated liver enzymes in the Netherlands. *J. Clin. Virol.* **2014**, *60*, 408-410, doi:10.1016/j.jcv.2014.05.009.
20. Scotto, G.; Martinelli, D.; Centra, M.; Querques, M.; Vittorio, F.; Delli Carri, P.; Tartaglia, A.; Campanale, F.; Bulla, F.; Prato, R.; Fazio, V. Epidemiological and clinical features of HEV infection: a survey in the district of Foggia (Apulia, Southern Italy). *Epidemiol. Infect.* **2014**, *142*, 287-294, doi:10.1017/s0950268813001167.
21. Jardi, R.; Crespo, M.; Homs, M.; van den Eynde, E.; Girones, R.; Rodriguez-Manzano, J.; Caballero, A.; Buti, M.; Esteban, R.; Rodriguez-Frias, F. HIV, HEV and cirrhosis: evidence of a possible link from eastern Spain. *HIV. Med.* **2012**, *13*, 379-383, doi:10.1111/j.1468-1293.2011.00985.x.
22. Riveiro-Barciela, M.; Buti, M.; Homs, M.; Campos-Varela, I.; Cantarell, C.; Crespo, M.; Castells, L.; Tabernero, D.; Quer, J.; Esteban, R.; Rodriguez-Frías, F. Cirrhosis, liver transplantation and HIV infection are risk factors associated with hepatitis E virus infection. *PLoS. One.* **2014**, *9*, e103028, doi:10.1371/journal.pone.0103028.
23. Đaković Rode, O.; Jemeršić, L.; Brnić, D.; Pandak, N.; Mikulić, R.; Begovac, J.; Vince, A. Hepatitis E in patients with hepatic disorders and HIV-infected patients in Croatia: is one diagnostic method enough for hepatitis E diagnosis? *Eur. J. Clin. Microbiol. Infect. Dis.* **2014**, *33*, 2231-2236, doi:10.1007/s10096-014-2187-7.
24. Ferreira, A.C.; Gomes-Gouvêa, M.S.; Lisboa-Neto, G.; Mendes-Correa, M.C.J.; Picone, C.M.; Salles, N.A.; Mendrone-Junior, A.; Carrilho, F.J.; Pinho, J.R.R. Serological and molecular markers of hepatitis E virus infection in HIV-infected patients in Brazil. *Arch. Virol.* **2018**, *163*, 43-49, doi:10.1007/s00705-017-3562-3.
25. Ramezani, A.; Velayati, A.A.; Khorami-Sarvestani, S.; Eslamifar, A.; Mohraz, M.; Banifazl, M.; Bidari-Zerehpoosh, F.; Yaghmaei, F.; McFarland, W.; Foroughi, M.; et al. Hepatitis E virus infection in patients infected with human immunodeficiency virus in an endemic area in Iran. *Int. J. STD. AIDS.* **2013**, *24*, 769-774, doi:10.1177/0956462413484457.
26. Rivero-Juarez, A.; Martinez-Dueñas, L.; Martinez-Peinado, A.; Camacho, A.; Cifuentes, C.; Gordon, A.; Frias, M.; Torre-Cisneros, J.; Pineda, J.A.; Rivero, A. High hepatitis E virus seroprevalence with absence of chronic infection in HIV-infected patients. *J. Infect.* **2015**, *70*, 624-630, doi:10.1016/j.jinf.2014.10.016.
27. Moss da Silva, C.; Oliveira, J.M.; Mendoza-Sassi, R.A.; Figueiredo, A.S.; Mota, L.D.D.; Nader, M.M.; Gardinali, N.R.; Kevorkian, Y.B.; Salvador, S.B.S.; Pinto, M.A.; Martinez, A.M.B. Detection and characterization of hepatitis E virus genotype 3 in HIV-infected patients and blood donors from southern Brazil. *Int. J. Infect. Dis.* **2019**, *86*, 114-121, doi:10.1016/j.ijid.2019.06.027.

28. Scotto, G.; Grisorio, B.; Filippini, P.; Ferrara, S.; Massa, S.; Bulla, F.; Martini, S.; Filippini, A.; Tartaglia, A.; Lo Muzio, L.; Fazio, V. Hepatitis E virus co-infection in HIV-infected patients in Foggia and Naples in southern Italy. *Infect. Dis. (Lond).* **2015**, *47*, 707-713, doi:10.3109/23744235.2015.1049658.
29. Bradley-Stewart, A.J.; Jesudason, N.; Michie, K.; Winter, A.J.; Gunson, R.N. Hepatitis E in Scotland: assessment of HEV infection in two high-risk patient groups with elevated liver enzymes. *J. Clin. Virol.* **2015**, *63*, 36-37, doi:10.1016/j.jcv.2014.11.025.
30. Zhou, S.; Ren, L.; Xia, X.; Miao, Z.; Huang, F.; Li, Y.; Zhu, M.; Xie, Z.; Xu, Y.; Qian, Y.; et al. Hepatitis E virus infection in HIV-infected patients: A large cohort study in Yunnan province, China. *J. Med. Virol.* **2018**, *90*, 1121-1127, doi:10.1002/jmv.25060.
31. Shahriarirad, R.; Erfani, A.; Rastegarian, M.; Zeighami, A.; Arefkhah, N.; Ghorbani, F.; Sarvari, J.; Sarkari, B. Seroprevalence of anti-hepatitis E antibodies and antigens among HIV-infected patients in Fars Province, southern Iran. *Virol. J.* **2020**, *17*, 109, doi:10.1186/s12985-020-01384-0.
32. Antonopoulou, N.; Schinas, G.; Kotsiri, Z.; Tsachouridou, O.; Protopapas, K.; Petrakis, V.; Petrakis, E.C.; Papageorgiou, D.; Tzimotoudis, D.; Metallidis, S.; et al. Testing Hepatitis E Seroprevalence among HIV-Infected Patients in Greece: The SHIP Study. *Pathogens.* **2024**, *13*, doi:10.3390/pathogens13070536.
33. Mbencho, M.N.; Hafza, N.; Cao, L.C.; Mingo, V.N.; Nyarko-Afriyie, E.; Achidi, E.A.; Ghogomu, S.M.; Velavan, T.P. Prevalence, genotype distribution, and risk factors of Hepatitis E virus in blood donors, HIV patients, and pregnant women in Southwest Cameroon. *Diagn. Microbiol. Infect. Dis.* **2025**, 111, 116748, doi:10.1016/j.diagmicrobio.2025.116748.
34. Politou, M.; Boti, S.; Androutsakos, T.; Valsami, S.; Pittaras, T.; Kapsimali, V. Seroprevalence of hepatitis E in HIV infected patients in Greece. *J. Med. Virol.* **2015**, *87*, 1517-1520, doi:10.1002/jmv.24214.
35. Kaba, M.; Richet, H.; Ravaux, I.; Moreau, J.; Poizot-Martin, I.; Motte, A.; Nicolino-Brunet, C.; Dignat-George, F.; Ménard, A.; Dhiver, C.; et al. Hepatitis E virus infection in patients infected with the human immunodeficiency virus. *J. Med. Virol.* **2011**, *83*, 1704-1716, doi:10.1002/jmv.22177.
36. Mateos-Lindemann, M.L.; Diez-Aguilar, M.; Galdamez, A.L.; Galán, J.C.; Moreno, A.; Pérez-Gracia, M.T. Patients infected with HIV are at high-risk for hepatitis E virus infection in Spain. *J. Med. Virol.* **2014**, *86*, 71-74, doi:10.1002/jmv.23804.
37. Junaid, S.A.; Agina, S.E.; Abubakar, K.A. Epidemiology and associated risk factors of hepatitis e virus infection in plateau state, Nigeria. *Virology. (Auckl).* **2014**, *5*, 15-26, doi:10.4137/vrt.S15422.
38. Caron, M.; Bouscaillou, J.; Kazanji, M. Acute risk for hepatitis E virus infection among HIV-1-positive pregnant women in central Africa. *Virol. J.* **2012**, *9*, 254, doi:10.1186/1743-422x-9-254.
39. Ng, K.P.; He, J.; Saw, T.L.; Lyles, C.M. A seroprevalence study of viral hepatitis E infection in human immunodeficiency virus type 1 infected subjects in Malaysia. *Med. J. Malaysia.* **2000**, *55*, 58-64.
